# Grain Weight and Taste Quality in *Japonica* Rice Are Regulated by Starch Synthesis and Grain Filling Under Nitrogen–Phosphorus Interactions

**DOI:** 10.3390/plants14030432

**Published:** 2025-02-01

**Authors:** Hongfang Jiang, Yanze Zhao, Liqiang Chen, Xue Wan, Bingchun Yan, Yuzhuo Liu, Yuqi Liu, Wenzhong Zhang, Jiping Gao

**Affiliations:** 1 Rice Research Institute, Shenyang Agricultural University, Shenyang 110866, China; jhf8870@stu.syau.edu.cn (H.J.); 2019200052@stu.syau.edu.cn (Y.Z.); 2021200066@stu.syau.edu.cn (X.W.); 2021200069@stu.syau.edu.cn (B.Y.); 2023200066@stu.syau.edu.cn (Y.L.); 2023200065@stu.syau.edu.cn (Y.L.); 2 School of Agriculture, Liaodong University, Dandong 118001, China; 923013@liaodongu.edu.cn

**Keywords:** grain filling, grain weight, nitrogen–phosphorus interactions, rice, starch synthesis, taste value

## Abstract

To reveal the regulatory effects of nitrogen and phosphorus interactions on grain-filling- and starch-synthesis-related enzymes, and grain weight of superior grains (SGs) and inferior grains (IGs) and taste quality, the *japonica* rice cultivar Shennong 265 was grown under field conditions with three nitrogen levels (210, 178.5, and 147 kg N ha^−1^; N3, N2, and N1) and two phosphorus levels (105 and 73.5 kg P ha^−1^; P2 and P1). At the N3 level, the yield of P1 was significantly lower (by 19.26%) compared to P2; at the N2 and N1 levels, P1 yielded higher than P2, peaking at N2P1. Spikelets per panicle showed P2 exceeding P1 at the same nitrogen level, with the highest for both SGs and IGs observed at N2P2, followed by N2P1. Reductions in nitrogen and phosphorus decreased the grain-filling rate but prolonged the duration for grain-filling. N2P1 maintained grain weight by extending the grain-filling duration across the early, middle, and late stages of IGs, and the middle and late stages of SGs. Increased nitrogen enhanced the activities of soluble starch synthase (SSS) and starch branching enzyme (SBE), whereas increased phosphorus inhibited these activities in SGs but enhanced them in IGs. Reduced nitrogen and phosphorus fertilizer diminished ADP glucose pyrophosphorylase (AGPP) and granule-bound starch synthase (GBSS) activities in SGs and IGs, inhibiting amylose accumulation while enhancing taste value. Compared with N3P2, the taste value of N2P1 increased significantly by 6.93%, attributed to a higher amylopectin/amylose ratio. N2P1 (178.5 kg N ha^−1^ and 73.5 kg P ha^−1^) optimized enzyme activity, starch composition, and grain filling, balancing both yield and taste, and thus demonstrated an effective fertilization strategy for stable rice production.

## 1. Introduction

Rice (*Oryza sativa* L.) is one of the most important food crops worldwide, accounting for a significant proportion of the energy, protein, and nutrient requirements for more than half of the world’s population [1]. With increased food production and improved living standards, rice consumption demand has shifted from simply meeting dietary needs to requiring high quality, thus highlighting the importance of rice quality alongside yield [2]. *Japonica* rice in northern China has a unique quality (especially taste) and yield advantage, and it has a particular competitive advantage in the international market. Nitrogen (N) and phosphorus (P) are key nutrients for rice growth and development and critical for sustaining rice yield and ensuring food security. However, to maximize rice yield, N and P fertilizers are usually applied above the minimum amount required to maintain maximum crop growth [3,4], resulting in prevalent N and P imbalances [5], which are more pronounced in China. The official recommendations in the northeastern rice cropping area are 120–180 kg ha^−1^ of N fertilizer and 37.5–75 kg ha^−1^ of P fertilizer. However, actual fertilization often exceeds the recommended amounts, leading to adverse effects, such as deteriorated rice quality, declining soil health, and environmental pollution [6,7,8]. Thus, sustainable and eco-friendly rice production is severely hindered in China. Therefore, reducing N and P inputs, achieving a fertilization balance, and maintaining stable yields are effective strategies for enhancing the production of high-quality flavorful rice.

The grain-filling capacity and starch content of rice grains determine the grain weight and quality [9] and are closely related to the position of spikelets on the panicle. In general, superior grains flower early and are located in the upper part of the rice spike, and they exhibit faster growth rates, shorter filling times, greater filling degrees, and higher grain weights. In contrast, inferior grains flower later and are located in the lower part of the spike, and they show slower growth rates, longer filling times, lower filling degrees, smaller grain weights, and even sterility [7,10]. Thus, inferior grains pose significant limitations to potential yield increases and quality improvements. Starch, the primary carbohydrate stored in rice grains, is crucial for determining rice quality, particularly taste [11]. Therefore, the grain-filling capacity of superior and inferior grains influences the extent of grain filling by affecting starch accumulation, which ultimately affects the grain weight and taste quality. Starch accumulation in grains depends mainly on the coordinated action of key enzymatic reactions involved in starch synthesis [12,13,14]. Therefore, enhancing the grain weight and balancing the amylose and amylopectin content by regulating the activities of key enzymes involved in starch synthesis during rice grain filling can improve the taste value.

In rice cultivation, excessive N application can lead to a low grain-filling rate and poor filling degree, resulting in loosely arranged starch grains [15,16] and reduced taste quality [1]. Conversely, appropriate N application can significantly increase the average grain-filling rate, maximum grain-filling rate, and grain weight during the active filling period of both superior and inferior grains, thereby enhancing rice yield [17,18]. However, increasing N application leads to an initially increasing and then decreasing trend in the maximum grain-filling rate of superior grains but an increasing trend in the maximum grain-filling rate of inferior grains [19]. Zhao et al. [20] reported that excessive N application reduces rice yield by inhibiting the filling of inferior grains. Additionally, N influences the composition of amylose and amylopectin by regulating the gene expression and enzyme activities of granule-bound starch synthase (GBSS), ISAs, and starch branching enzyme (SBE) [21,22]. Appropriate P fertilization enhances the activities of sucrose synthase and SBE, promotes sucrose degradation, and facilitates starch synthesis and accumulation [23]. Li et al. [24] reported that increased application of P fertilizer increased the activity of enzymes related to starch synthesis in the superior and inferior grains of different low P-tolerant rice varieties. Therefore, the application and balance of N and P fertilizers influence the grain-filling ability and key starch synthesis enzyme activities at different grain positions. This is expected to improve rice taste quality and grain weight by regulating the amylose and amylopectin content, thereby achieving a synergistic enhancement of yield and quality. However, most previous studies have focused on individual nitrogen or phosphorus fertilizer levels rather than a range of values.

To date, limited research has focused on how nitrogen-phosphorus interactions regulate the filling characteristics of superior and inferior rice grains, the activities of key enzymes involved in starch synthesis, and how these factors are related to taste quality and grain weight. In this study, the effects of three N levels and two P levels on the grain-filling dynamics, key starch synthesis enzyme activities, grain weight, and starch content of superior and inferior grains, and taste quality were investigated under field conditions. The main objectives of this study were to (1) investigate the differences in nitrogen–phosphorus interactions that affect the filling process of superior and inferior grains and their effects on grain weight and yield; (2) identify variations in the activities of key enzymes involved in starch synthesis in superior and inferior grains and their regulatory effects on starch content under different N and P levels; and (3) explore the relationship between grain weight and taste quality and the filling and starch synthesis of superior and inferior grains. This study aimed to provide a theoretical basis for balanced N and P fertilization to achieve stable yield and high-quality *japonica* rice production in northern China.

## 2. Results

### 2.1. Yield, Spikelets per Panicle, and Grain Weight

**Figure 1:**
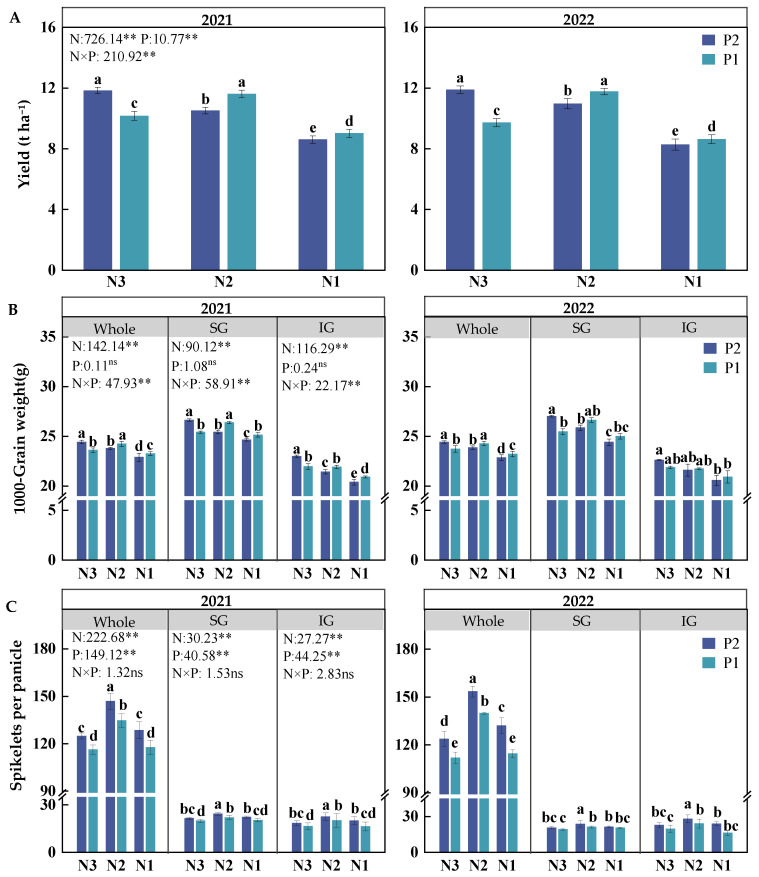
Effects of nitrogen–phosphorus interactions on rice yield (**A**), 1000-grain weight (**B**), and spikelets per panicle (**C**). SG and IG represent superior and inferior grains, respectively; N3, N2, and N1 represent N applications of 210, 178.5, and 147 kg ha^−1^, respectively; P2 and P1 represent P applications of 105 and 73.5 kg ha^−1^, respectively. Bars represent ±SE of the mean. Different letters on top of the histograms indicate a significant difference at *p* < 0.05 between the different treatments in the same year; ** indicates that the effects of variables are significant at 0.01 levels; and ns indicates no significant effect.

The application of N and P fertilizers and their interactions significantly affected the yield (*p* < 0.01, Figure 1A), with a consistent pattern observed over both years. N3P2 produced the highest yield, exceeding that of N3P1 by 19.26% in the two-year average. At the N2 level, the yield of P1 was significantly higher than that of P2, although this increase was not significant compared to that of N3P2. Compared to N3P2, the two-year average yields of N1P2 and N1P1 decreased by 28.84% and 25.63%, respectively.

The 1000-grain weight was significantly influenced by the N level and its interaction with the P level (*p* < 0.01, Figure 1B). N3P2 exhibited the highest 1000-grain weight, surpassing that of N3P1 by 3.15% in the two-year average. Specifically, the weights of superior grains (SGs) and inferior grains (IGs) in N3P2 increased by 5.44% and 4.03%, respectively. At the N2 and N1 levels, the 1000-grain weight of P1 was higher than that of P2. N2P1 achieved the highest 1000-grain weight, although the value was not significantly different from that of N3P2. Additionally, the difference in 1000-grain weight between N2P1 and N3P2 was more pronounced for IGs than for SGs.

The N and P fertilizer levels had statistically significant effects on spikelets per panicle (*p* < 0.01; Figure 1C). At the same N level, the spikelets per panicle in P2 was significantly higher than that in P1, with the N2P2 combination achieving the highest spikelets per panicle, followed by N2P1. Compared to N3P2, N2P2 demonstrated a 20.81% increase in spikelets per panicle on average in two years, with significant increases of 15.17% and 22.41% in SGs and IGs, respectively. For N2P1, the number of spikelets per panicle increased by 10.43% in the two-year average, with SGs and IGs increasing by 3.50% and 7.20%, respectively; however, these differences were not statistically significant.

**Figure 2:**
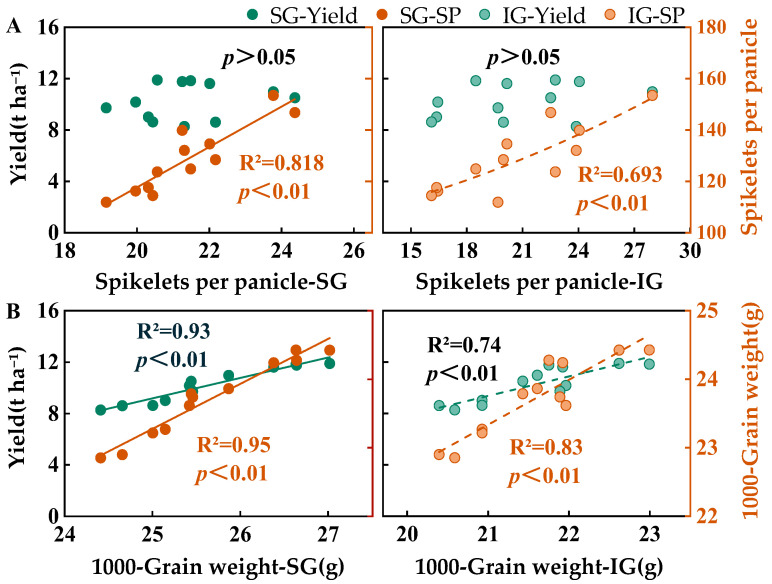
Relationship between the spikelets per panicle and yield (**A**) and 1000-grain weight and yield (**B**). SG and IG represent superior grain and inferior grains, respectively; when *p* > 0.05, the fitting model cannot provide statistical significance evidence, so there are no invalid fitting lines in the figures; R^2^ represents coefficient of determination.

The spikelet numbers in SGs and IGs were significantly positively correlated with the spikelets per panicle (*p* < 0.01; Figure 2A). The influence of SGs on the spikelets per panicle was greater than that of IGs (R² = 0.82, SGs; R² = 0.70, IGs). However, there was no significant correlation between the number of spikelets in SGs and IGs and yield. The grain weights of SGs and IGs showed a positive linear correlation with the yield and 1000-grain weight, with the correlations attaining highly significant levels (*p* < 0.01; Figure 2B). Additionally, the correlation between the yield and 1000-grain weight was more pronounced for SGs than for IGs.

### 2.2. Grain-Filling Characteristics of Superior Grains and Inferior Grains

**Figure 3:**
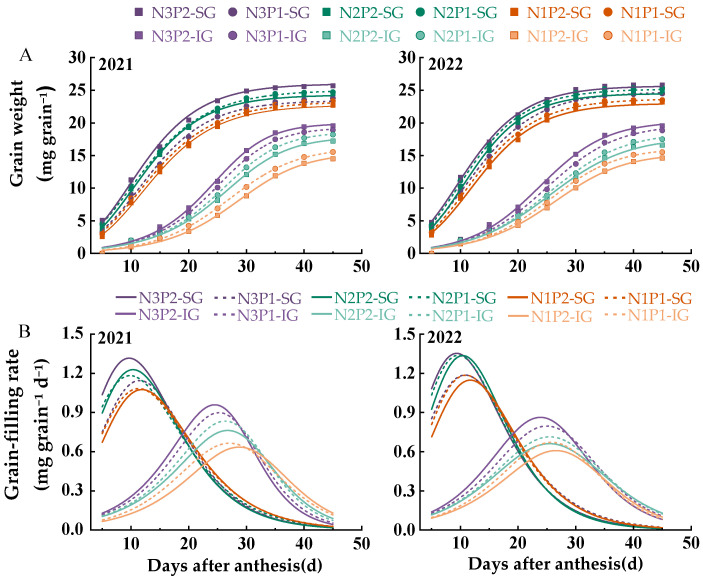
Effects of nitrogen–phosphorus interactions on grain weight (**A**) and grain-filling rate (**B**) in rice. SG and IG represent superior and inferior grains, respectively; N3, N2, and N1 represent N applications of 210, 178.5, and 147 kg ha^−1^, respectively; P2 and P1 represent P applications of 105 and 73.5 kg ha^−1^, respectively.

Compared to the IGs, the SG grain weight increased rapidly after anthesis (Figure 3). N3P2 had the highest final grain weight for both SGs and IGs. N3P1 significantly reduced the grain weight of SGs but led to the second-highest grain weight in IGs after N3P2. In contrast, N1P2 and N1P1 reduced the grain weight of SGs and IGs, with N1P2 having the most pronounced effect.

The weight gain processes of SGs and IGs under different N and P levels were modeled using the Richards growth equation (Figure 3A), and its derivative was used to obtain the grain-filling rate curve (Figure 3B). The coefficients of determination (R²) for the fitted curves of each treatment were above 0.95 (Table 1), indicating that the Richards equation effectively modeled the grain-filling process for both SGs and IGs in each treatment. According to Figure 3 and Table 1, the initial grain-filling potential (R_0_), maximum grain-filling rate (Gmax), and mean grain-filling rate (Gmean) of SGs were generally higher than those of IGs, indicating a faster grain-filling process and higher grain weight. The grain-filling process of IGs was significantly delayed, and the grain-filling rate of IGs increased rapidly only after the grain-filling rate of SGs began to decrease, resulting in a longer time for Gmax (Tmax.G) and active grain-filling period (D) compared to SGs.

Different levels of N and P affected the filling parameters of SGs and IGs (Table 1). N3P2 led to the highest R_0_ for SGs, with a 72.5% increase over N3P1 in the two-year average. Similarly, the R0 of N2P1 was slightly lower than that of N3P2, with a 35.41% increase over that of N2P2. The N1 level decreased the R0 of SGs, especially in N1P2. For IGs, R_0_ followed the order N3 > N2 > N1. At the N3 level, P2 exceeded P1. However, this trend was reversed at the N2 and N1 levels. A reduction in N levels decreased Gmax, weight at Gmax (Wmax), and Gmean in both SGs and IGs while prolonging Tmax.G and D. In SGs, at N3 level, P1 decreased Gmax, Wmax, and Gmean but increased Tmax.G and D by approximately 1.47–1.83 and 1.13–2.83 d, respectively. At the N2 level, Gmax, Wmax, and Gmean were slightly lower for P1 than for P2; however, Tmax.G was reduced by 0.51–0.78 d while D was prolonged by approximately 0.67–2.01 d, and there were minimal differences in these parameters between N1P1 and N1P2. Regarding IGs, Gmax, Wmax, and Gmean were the highest in N3P2, followed by N3P1. In addition, Tmax.G and D were shortened by approximately 0.75–1.09 and 1.02–1.98 d in N3P2 compared to N3P1. At the N2 and N1 levels, Gmax, Wmax, and Gmean were higher for P1 than for P2, with little difference in Tmax.G and D between the two treatments. The Gmax, Wmax, and Gmean values of N2P2 were slightly lower than those of N3P2 and N3P1. However, Tmax.G was prolonged by 0.70–1.06 d compared to that of N3P2.

**Figure 4:**
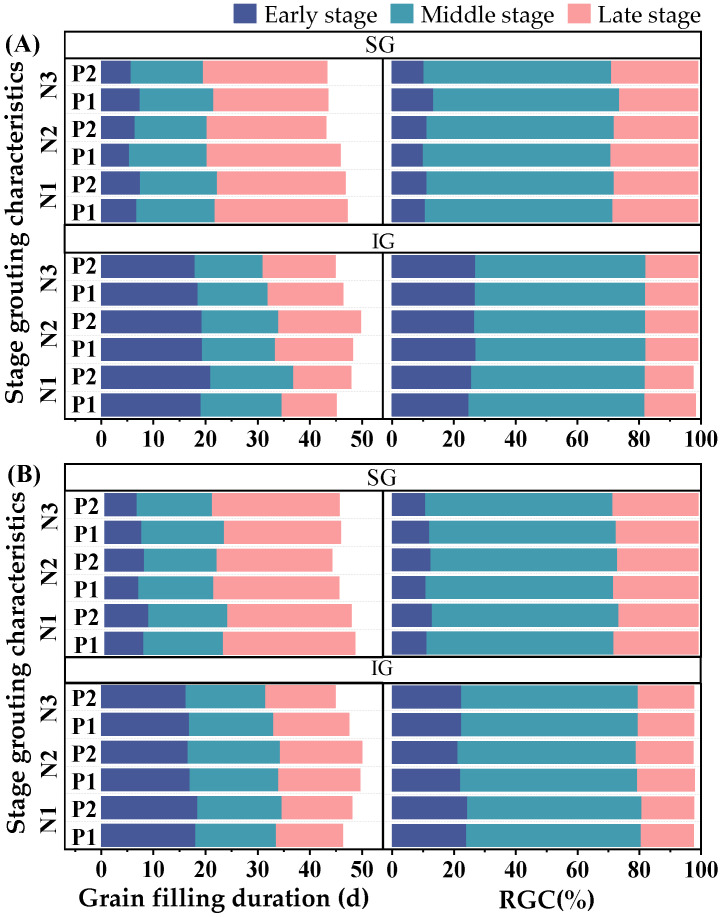
Effects of nitrogen–phosphorus interaction on characteristics of grain filling in the early, middle, and late growth stages ((**A**)—2021, (**B**)—2022). N3, N2, and N1 represent N application of 210, 178.5, and 147 kg ha^−1^, respectively; P2 and P1 represent P application of 105 and 73.5 kg ha^−1^, respectively; RGC, the grain-filling contribution rate; SG, superior grains; IG, inferior grains.

The grain-filling process was categorized into three stages: early, middle, and late (Figure 4). In SGs, the grain-filling duration was ordered late > middle > early. The grain-filling contribution rate (RGC) was highest in the middle stage, followed by the late stage, and lowest in the early stage. At the N3 level, the grain-filling duration in the early and middle stages of P2 was shortened, and the grain-filling duration was prolonged in the later stages compared with that of P1. Concurrently, the RGC decreased in the early stage but improved in the late stage. At the N2 and N1 levels, the reduction in P fertilizer shortened the grain-filling duration and decreased the RGC in the early stage, prolonged the grain-filling durations in the middle and late stages (especially at the N1 level), and increased the RGC in the late stage. There was little difference in the RGC of the middle stage among the treatments.

In IGs, reductions in N and P fertilizers tended to prolong the grain-filling duration in the early and middle stages. This effect was more pronounced at the N2 level, whereas N1 shortened the grain-filling duration in the later stages. At the N3 level, significant differences in the RGC were not observed between each stage. At the N2 level, P1 increased the RGC in the early stage but shortened the grain-filling duration in the middle and late stages, with minimal variation in the RGC at these stages. At the N1 level, the reduction in P fertilizer slightly decreased the RGC in the early stage. However, it increased the RGC in the middle and late stages. Overall, compared with N3P2, N2P1 shortened the grain-filling duration in the early stage of SGs but prolonged the grain-filling duration in the middle and late stages. At the same time, N2P1 prolonged the grain-filling duration at all stages of IGs.

### 2.3. Starch Content and Taste Value in Rice

Both N and P fertilizers had significant effects on the amylose content of rice (Table 2, *p* < 0.01). The amylopectin content, total starch content, and taste value were primarily influenced by N and its interaction with P fertilizer (*p* < 0.01). Overall, N and P reductions tended to decrease the amylose content and improve the taste quality of rice. Compared with N3P2, N3P1 decreased the amylose, amylopectin, total starch content, and taste value by 2.36%, 7.92%, 6.41%, and 3.36% on average in two years, respectively, with the differences in amylopectin and total starch content reaching significance. In addition, the taste values of N2P1 and N2P2 were significantly increased by 6.93% and 2.93% on average in two years, respectively. At the N1 level, both P fertilizer treatments reduced amylose, amylopectin, and total starch content, and the taste value was significantly lower than that of N3P2 (except for N1P1 in 2022, where the difference in taste value was not significant), especially in N1P2.

### 2.4. Changes in the Enzyme Activities of ADPG, GBSS, SSS, and SBE in SGs and IGs

**Figure 5:**
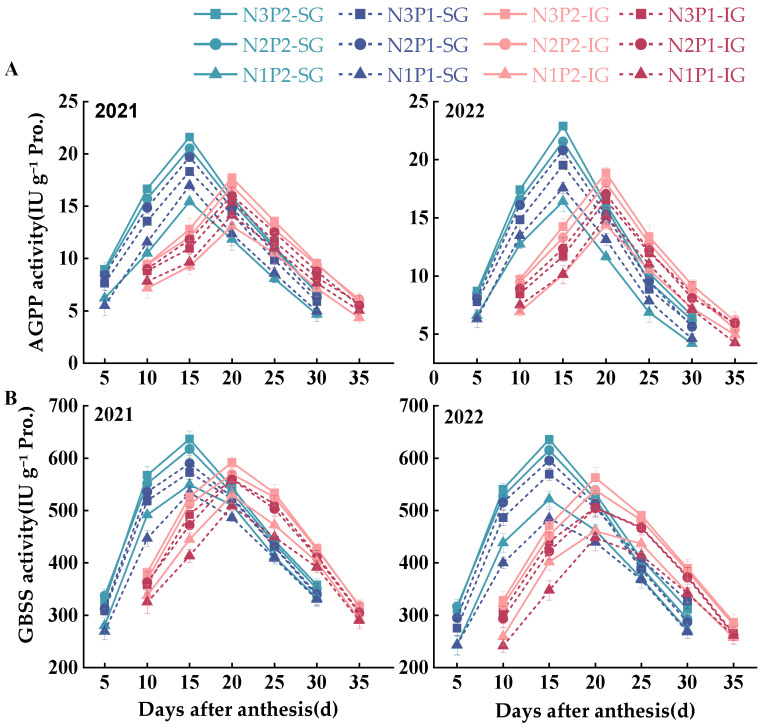
Effects of nitrogen–phosphorus interaction on the enzyme activities of ADP glucose pyrophosphorylase (AGPP) and granule-bound starch synthase (GBSS) in SGs and IGs during the filling stage ((**A**)—AGPP, (**B**)—GBSS). N3, N2, and N1 represent N application of 210, 178.5, and 147 kg ha^−1^, respectively; P2 and P1 represent P application of 105 and 73.5 kg ha^−1^, respectively.

**Figure 6:**
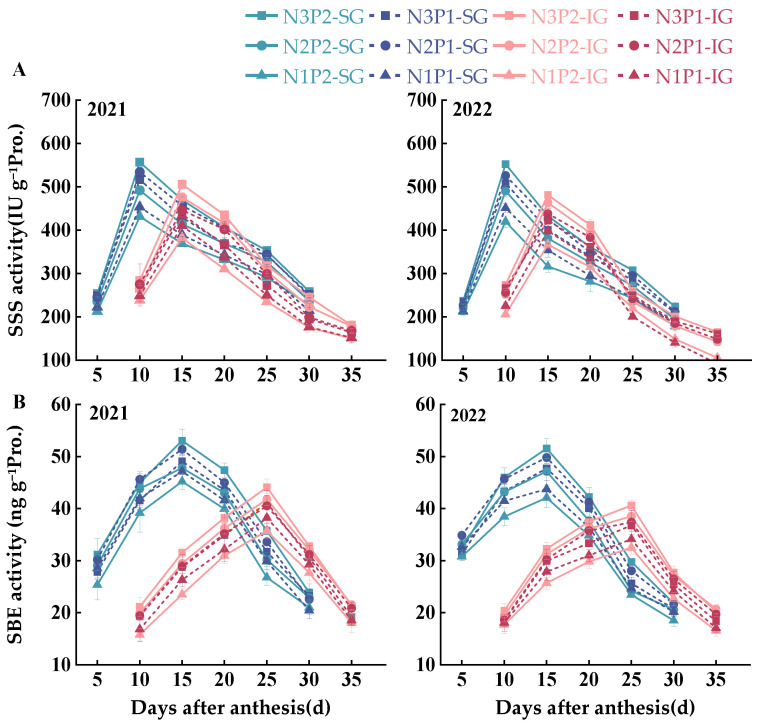
Effects of nitrogen–phosphorus interaction on the enzyme activities of soluble starch synthase (SSS) and starch branching enzyme (SBE) in SGs and IGs during the filling stage ((**A**)—SSS, (**B**)—SBE). N3, N2, and N1 represent N application of 210, 178.5, and 147 kg ha^−1^, respectively; P2 and P1 represent P application of 105 and 73.5 kg ha^−1^, respectively.

The activities of the key enzymes for starch synthesis all showed a single peak as the grain-filling process progressed, increasing initially and then decreasing rapidly or gradually (Figure 5 and Figure 6); however, the timing of the peak activity varied, occurring either early or late.

As shown in Figure 6, both the ADP glucose pyrophosphorylase (AGPP) and GBSS activities peaked at 15 and 20 d after anthesis in SGs and at 20 d in IGs. The AGPP and GBSS activities were higher in SGs and IGs for P2 than P1 at both the N3 and N2 levels. At the N1 level, both P treatments significantly reduced the AGPP and GBSS activities in SGs and IGs, with N1P2 presenting the lowest AGPP activity in SGs and IGs and N1P1 presenting the lowest GBSS activity in SGs and IGs. Overall, N3P2 had the highest AGPP and GBSS activities in SGs and IGs, followed by N2P2 and N2P1. N2P1 had lower GBSS activities in IGs than N3P1, but the difference between the two was not significant.

The soluble starch synthase (SSS) activity in SGs increased rapidly with the onset of filling, peaking at 10 d after anthesis, whereas the activity in IGs peaked at 15 d after anthesis (Figure 6A). The SSS activity in SGs and IGs gradually decreased with N reduction at P2; however, at P1, the SSS activity followed the order N2 > N3 > N1. At the N3 level, the SSS activity in both SGs and IGs of P2 was significantly higher than that of P1 at their respective peaks. At the N2 level, the SSS activity of P2 in SGs was higher than that of P1, whereas the activity of P2 in IGs was lower than that of P1. P fertilizer reduction significantly increased SSS activity in SGs and IGs at the N1 level. Overall, the SSS activity in SGs was highest at N3P2, followed by N2P1 and N3P1, whereas the activity in IGs was highest at N3P2, followed by N2P2 and N2P1.

The SBE activity of SGs peaked at 15 d after the anthesis and then gradually decreased (Figure 6B), and the activity of IGs peaked at 20 d after the anthesis. At P2, the SBE activity in both SGs and IGs gradually decreased with N reduction. At P1, the SBE activity in SGs decreased in the order of N2 > N3 > N1, and the activity in IGs decreased progressively with N reduction. At the N3 level, SBE activity in both SGs and IGs was higher in P2 than in P1. P fertilizer reduction increased SBE activity in SGs but decreased it in IGs at the N2 and N1 levels. In summary, the N3P2 treatment had the highest SBE activity in both SGs and IGs, while the N2P1 treatment did not cause significant differences in activity in SGs relative to that in N3P2 but led to significantly lower activity in IGs relative to that in N3P2.

### 2.5. Grain Filling Characteristics, Starch Synthase Activity in Relation to Starch Content, Taste Value, and Grain Weight

**Figure 7:**
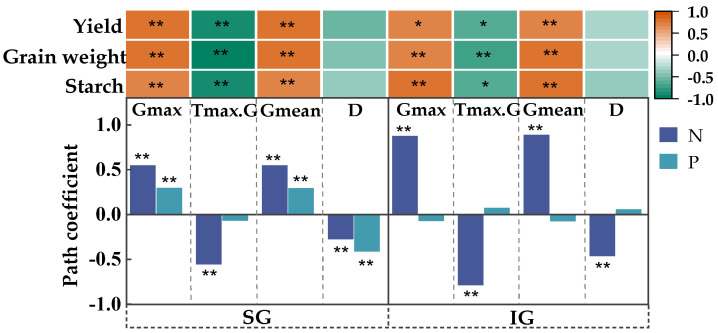
Response of grain-filling characteristics to N and P and their correlation with the starch content, grain weight, and yield. * and ** indicate that the effects of variables are significant at 0.05 and 0.01 levels.

The responses of the grain-filling characteristics to N and P and their correlations with the starch content, grain weight, and yield were analyzed (Figure 7). For SGs, N and P levels showed significant positive effects on the Gmax and Gmean in SGs (path coefficients of 0.55 and 0.29, respectively), but had significant negative effects on the D. Tmax.G was significantly negatively influenced by N level alone. Moreover, Tmax.G showed a significant negative correlation with starch content, grain weight, and yield.

The grain-filling characteristics of IGs were primarily influenced by N fertilization, which significantly positively affected the Gmax and Gmean of IGs. Gmax and Gmean were significantly and positively correlated with the starch content, grain weight, and yield. Conversely, N fertilization significantly negatively impacted Tmax.G and D. Tmax.G was significantly negatively correlated with the yield and starch content (*p <* 0.05) and negatively correlated with grain weight (*p <* 0.01).

**Figure 8:**
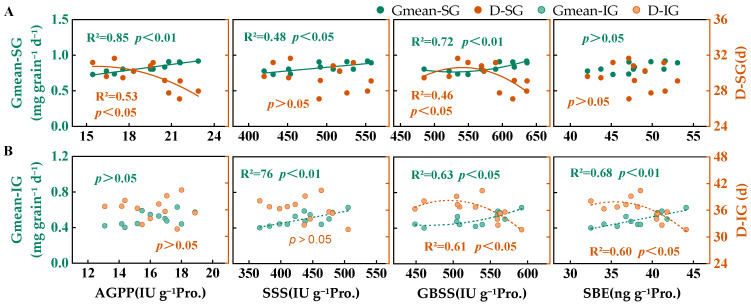
Correlation between the peak activities of AGPP, SSS, GBSS, and SBE with the mean grain-filling rate (Gmean) and active grain-filling period (D) ((**A**)—Superior grains, SG; (**B**)—Inferior grains, IG). When *p* > 0.05, the fitting model cannot provide statistical significance evidence, so there are no invalid fitting lines in the figures; R^2^ represents coefficient of determination.

As shown in Figure 8A, the Gmean of SGs was significantly affected by its AGPP and GBSS activities (*p* < 0.01) as well as by its SSS activity (*p* < 0.05). D was significantly influenced by the AGPP and GBSS activities. Specifically, D decreased with increasing AGPP activity, and within a certain range, D increased with increasing GBSS activity. However, beyond this range, additional increases in GBSS activity did not further increase D. As illustrated in Figure 8B, the Gmean of IGs showed a significant positive correlation with both the SSS and SBE activities (*p* < 0.01) and GBSS activities (*p* < 0.05). Furthermore, the D of IGs was significantly influenced by the GBSS and SBE activities (*p* < 0.05). Excessive GBSS and SBE activity shortened the D of IGs.

**Figure 9:**
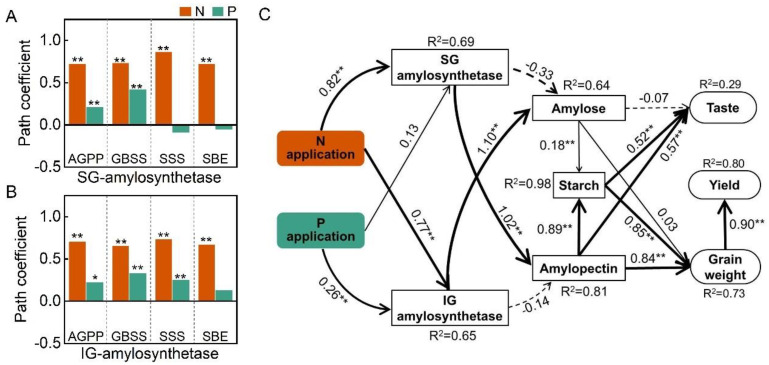
Structural equation model analysis (SEM) showing the effects of nitrogen and phosphorus on starch synthase activity, starch content, taste value, 1000-grain weight, and yield (**C**), path coefficient of SG (**A**) and path coefficient of IG (**B**). The R^2^ values denote the proportions of variance explained by relationships with other variables in SEM. Single-headed arrows indicate the hypothesized direction of causation. Indicated values denote the standardized path coefficients of a positive (positive values) or negative (negative values) effect. Solid and dashed lines represent positive and negative paths, respectively. The thickness of the black line indicates the strength of the causal relationship. * and ** represent *p* < 0.05 and *p* < 0.01, respectively.

Structural equation model (SEM) analysis (Figure 9) showed that N and P levels regulate amylose and amylopectin content by influencing the activities of key enzymes involved in starch synthesis in SGs and IGs, influencing rice taste value, grain weight, and yield. N fertilizer had a significant positive effect on the activities of key enzymes involved in starch synthesis in both SGs and IGs (Figure 9A,C), whereas P fertilizer positively influenced only the activities of AGPP and GBSS in SGs and AGPP, GBSS, and SSS in IGs.

The activity of key starch-synthesizing enzymes in SGs had a significant positive effect on the amylopectin content (*p* < 0.01), whereas the activity of key starch-synthesizing enzymes in IGs had a significant positive impact on the amylose content (*p* < 0.01). Both amylose and amylopectin contributed significantly to the total starch content (path coefficient: amylopectin = 0.89 > amylose = 0.18). The significant positive effect of starch content on the taste value was primarily due to the influence of the amylopectin content. The starch content significantly positively affected grain weight, which was attributed to the regulatory effect of amylopectin content. Finally, grain weight had a significant positive effect on yield.

## 3. Discussion

### 3.1. Effects of N and P on Both the Yield and Grain Weight and Their Relationship with Grain-Filling Characteristics

Chemical fertilizers are important in increasing grain yield and ensuring food security. The effectiveness of fertilizer application in achieving high-yield and high-quality rice depends on optimizing fertilizer management practices [25]. Balanced application of nitrogen and phosphorus fertilizers is essential for rice yield, and consistently balanced application can enhance soil phosphorus availability, increase rice phosphorus uptake, and improve yield [26]. Numerous studies have shown that rice yield increases with increasing nitrogen application within a certain range, beyond which excessive nitrogen can reduce yield [27,28,29,30]. Similarly, excessive phosphorus fertilizer has not been shown to significantly improve agronomic traits [31,32] and can be detrimental to rice yield [33,34]. Lu et al. [35] reported that a soil Olsen–P content of 5–7 mg ha^−1^ was sufficient to meet the high-yield requirements of rice, and no yield increase was observed at phosphorus application rates over 60 mg ha^−1^ [36]. In our experimental area, the soil available phosphorus content was 14.3 mg ha^−1^, and the results showed differences in the amount of applied phosphorus required to achieve the maximum yield under different nitrogen application levels. Under conventional nitrogen levels (210 kg ha^−1^), a 30% reduction in phosphorus fertilizer (73.5 kg ha^−1^) significantly reduced rice yield. High nitrogen levels promoted spikelet occurrence in high tillers of rice, thereby negatively affecting spikelets per panicle, particularly in IGs. However, yield was significantly higher at 73.5 kg ha^−1^ phosphorus compared to 105 kg ha^−1^ phosphorus combined with 15% (178.5 kg ha^−1^) and 30% (147 kg ha^−1^) nitrogen fertilizer reduction. Zhou et al. [37] reported that excessive nitrogen application increased the degradation of primary and secondary spikelets, which decreased the “storage capacity” of rice. In the current study, however, nitrogen application of 178.5 kg ha^−1^ inhibited small tiller occurrence at high positions and significantly increased the spikelets per panicle, contributing to the stable yield of N2P1. Previous studies have identified a synergistic regulatory mechanism between nitrogen and phosphorus [38]. For example, the rice nitrogen sensor NRT1.1B interacts with the phosphorus sensor SPX4 to activate the expression of nitrate-responsive and phosphorus-starvation-induced genes mediated by nitrate signaling molecules, thereby enhancing the synergistic utilization of nitrogen and phosphorus nutrients [39]. Overall, the excessive application of nitrogen and phosphorus fertilizers does not effectively improve rice agronomic traits and yield performance relative to sustainable agricultural practices, and it increases environmental risks [16,25,40]. Our study showed that appropriate reductions of nitrogen and phosphorus application achieved stable yields over two years by increasing the spikelets per panicle, especially for IGs, and consistently maintaining the 1000-grain weight.

The results showed a highly significant positive correlation between the grain weight of both SGs and IGs and yield (Figure 2). The levels of nitrogen and phosphorus fertilizer inputs in rice paddies influence the “storage capacity” of the rice plants and the plumpness of the grains through the grain-filling process. Previous studies have shown that overapplication of nitrogen reduces the grain-filling efficiency of rice, whereas underapplication significantly reduces grain yield [41]. Compared with high nitrogen levels, conventional nitrogen application significantly increases the grain-filling rate and weight of IGs [42]. Currently, few studies have focused on the effects of nitrogen and phosphorus fertilizer interactions on the filling of SGs and IGs in *japonica* rice. Our study showed that nitrogen and phosphorus fertilizer reduction reduced the R_0_ and grain-filling rate of SGs and IGs but tended to prolong the active grain-filling period. However, at the 147 kg ha^−1^ nitrogen fertilizer level, prolonging the active grain-filling period was insufficient to compensate for the loss from the reduced grain-filling rate, resulting in a reduction in grain weight for both SGs and IGs. At the 178.5 kg ha^−1^ nitrogen fertilizer level, although the filling rate of SGs and IGs was slightly lower than that of conventional fertilization, it prolonged the grain-filling duration in the middle and late stages of SGs and maintained the grain-filling contribution rate of each stage. Simultaneously, it prolonged the grain-filling duration of the IGs in the early, middle, and late stages, thereby maintaining grain weight. In contrast, the reduction in nitrogen and phosphorus fertilizers in this study increased the spikelets per panicle, which intensified the competition for assimilates amidst enhanced “storage capacity”, which resulted in decreased grain filling, especially affecting the weight of IGs. Correlation analysis showed (Figure 7) that nitrogen and phosphorus positively influenced the filling rate and grain weight of SGs and IGs. The active grain-filling period of SGs was negatively regulated by nitrogen and phosphorus. However, the active grain-filling period of inferior grains was significantly affected primarily by nitrogen fertilizer. In contrast, the effect of phosphorus fertilizer was not significant. Therefore, under the conditions of this study, appropriate reduction in nitrogen and phosphorus fertilizers could compensate for the loss of low grain-filling rates by extending the active grain-filling period of both SGs and IGs. However, insufficient nitrogen levels inhibit grain filling and limit increases in potential yield. Furthermore, we observed that the active grain-filling period of SGs in 2022 was shorter than in 2021, whereas the active grain-filling period of IGs was longer. This difference may be attributed to the higher average temperature in early August 2022 compared to 2021, which accelerated the filling of SGs. Conversely, the lower average temperature in mid- and late August 2022 extended the active grain-filling period of IGs.

In addition, studies have reported that the concentration of the assimilated substrate in grains is not the primary limiting factor for the slow and poor filling rates of IGs. Instead, low starch synthesis efficiency is likely the main factor underlying the poor rates [43,44]. SuSase, AGPP, SSS, and SBE are key enzymes involved in the sucrose–starch metabolism pathway, and they are closely related to the grain-filling rate [45,46]. According to Fu et al. [46], the low activities of SuSase, AGPP, and SSS during the filling process in super rice are the major contributors to the low filling rate and reduced grain weight of IGs. Our results (Figure 8) indicated that in SGs, the activities of AGPP, SSS, and GBSS were significantly positively correlated with the grain-filling rate. AGPP and GBSS activities significantly affected the active grain-filling period, suggesting that the low activities of AGPP, SSS, and GBSS are key factors in the reduced grain-filling rate and light weight of SGs. In contrast, the SSS, GBSS, and SBE activities in IGs showed significant positive correlations with the grain-filling rate. However, the active grain-filling period of IGs was first prolonged and then shortened with GBSS and SBE activities, indicating that excessively high GBSS and SBE activities in IGs would be detrimental to the filling of IGs.

### 3.2. Effects of Nitrogen–Phosphorus Interactions on Rice Quality and the Relationship with Enzymes Related to Starch Synthesis

Starch is the primary storage carbohydrate that influences rice quality [47,48]. Both the amylose [49,50,51] and amylopectin contents [52,53] are closely related to the taste quality of rice. Rice with superior taste tends to have a lower amylose content [54]. Previous studies have shown that high nitrogen fertilizer application increases the amylose content of rice but decreases the taste and cooking quality [1]. Nitrogen and phosphorus fertilizers reduce the taste value of rice by increasing its hardness and viscosity [55]. Appropriate application of phosphorus fertilizer promotes starch degradation, reduces hardness, and improves viscosity and taste, while excessive phosphorus can increase hardness, lower taste value, and degrade food quality [56]. Our results showed that reduced nitrogen and phosphorus fertilizers tended to lower the amylopectin content in rice, although the responses of amylopectin and total starch content to phosphorus fertilizer varied based on the nitrogen levels. At 210 kg N ha^−1^, reduced phosphorus application significantly decreased amylopectin content, resulting in significantly lower starch content and taste quality; at 178.5 kg N ha^−1^ and 147 kg N ha^−1^, reduced phosphorus fertilization increased the amylopectin and amylose content but improved the taste quality, with these effects diminishing at 147 kg N ha^−1^. From a structural perspective, nitrogen fertilizers can increase the proportion of long amylopectin chains [57]. A high proportion of long-chain amylopectin increases the relative crystallinity of starch [58] and binds to protein, thereby limiting the swelling of the starch and leading to poorer rice taste quality. Structural equation model analysis (Figure 9) showed that taste quality was significantly and positively correlated with the amylopectin content and negatively correlated with the amylose content. In this study, N2P1 significantly improved the taste quality, mainly by increasing the amylopectin/amylose ratio.

Starch biosynthesis in the plant endosperm involves a series of key enzymes working in coordination [59]. AGPP is crucial in synthesizing adenosine diphosphate glucose (the primary substrate of starch synthesis) and is considered the rate-limiting enzyme in this pathway [60]. Our results showed that nitrogen and phosphorus fertilizers significantly affected AGPP activity in both SGs and IGs, with nitrogen having a significantly greater effect, especially in the IGs. Within the range of 147–210 kg N ha^−1^, decreasing nitrogen levels tended to decrease AGPP activity in both the SGs and IGs. The influence of phosphorus fertilizer on AGPP activity depended on the different nitrogen application levels. Specifically, at nitrogen rates of 210 kg N ha^−1^ and 178.5 kg N ha^−1^, reducing phosphorus fertilizer led to decreased AGPP activity, with a smaller differential effect at 178.5 kg N ha^−1^, particularly in IGs. Conversely, reducing the phosphorus levels mitigated the decrease in AGPP activity under low nitrogen conditions. Ma et al. [56] reported that OsPHO1;2 could promote the activity of starch-synthesis-related enzymes, such as AGPase, by regulating the Pi content in the endosperm under low-phosphorus conditions, thereby maintaining grain filling and starch content. SSS catalyzes the formation of amylopectin α-1,4 glycosidic bonds [61], while SBE is a key enzyme promoting amylopectin formation [62]. In our study, nitrogen fertilizer had significant positive effects on SSS and SBE activities in both SGs and IGs, but phosphorus fertilizer had a significant positive effect on SSS activity only in IGs. Therefore, within the range of 147 kg N ha^−1^ to 210 kg N ha^−1^ and 73.5 kg P ha^−1^ to 105 kg P ha^−1^, increasing nitrogen was beneficial for increasing SSS and SBE activity in SGs, while increasing phosphorus improved the activity of SSS and SBE in IGs but inhibited their activity in SGs, thereby negatively affecting amylopectin accumulation in the grains. GBSS activity, which is closely related to amylose synthesis [24], was increased by nitrogen treatment, which increased the amylose content [63]. Our study showed that nitrogen and phosphorus fertilizers had a significant positive effect on the GBSS activity of both SGs and IGs, suggesting that excessive application of nitrogen and phosphorus fertilizers could increase the GBSS activity of both SGs and IGs and promote amylose accumulation, thereby reducing rice taste. Consequently, a balanced application of nitrogen and phosphorus fertilizers can coordinate the activities of key enzymes for starch synthesis in strong and weak grains during the filling period, effectively regulate starch content, and contribute to the cultivation of high-quality rice.

## 4. Materials and Methods

### 4.1. Plant Materials and Growth Conditions

**Figure 10:**
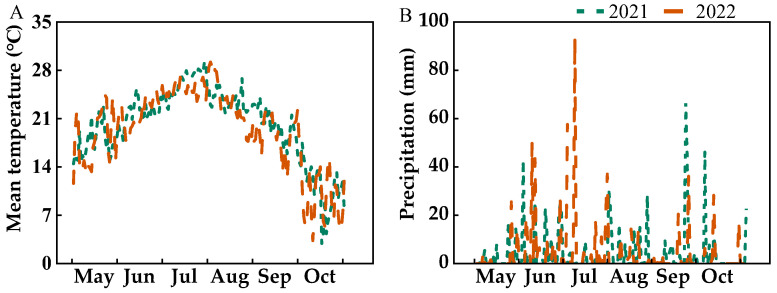
Mean temperature (**A**) and precipitation (**B**) during the rice growing seasons of 2021 and 2022.

The studies were conducted at the Kalima Rice Experimental Station of Shenyang Agricultural University (41°51′ N, 122°71′ E) in Liaoning Province, China, during 2021 and 2022. The soil was sandy loam and contained 16.9 g kg^−1^ of soil organic matter, 0.41 g kg^−1^ of total nitrogen, 92.7 mg kg^−1^ of alkali-N, 0.98 g kg^−1^ of total phosphorus, 14.3 mg kg^−1^ of Olsen–P, and 64.9 mg kg^−1^ of available K in the 0–20 cm tillage layer, with a pH of 6.09. The location is a low altitude rice growing area with an altitude of 17 m, belonging to a subtropical monsoonal climate, with an accumulated temperature range of 3350–3450 °C and a frost-free period of 185 d. Figure 10 illustrates the daily mean temperature and precipitation during the rice-growing seasons of 2021 and 2022. The tested variety was Shennong 265 (*japonica* rice), which has 15 leaves on its main stem and a growth period of approximately 158 d. This variety was developed by the Rice Research Institute at Shenyang Agricultural University and is the first *japonica* super rice variety produced since the launch of the “China Super Rice Breeding” project. It exhibits strong stress resistance, particularly exceptional resistance to rice blast disease, and demonstrates high adaptability to diverse planting conditions. The nitrogen fertilizers were urea (46% N) and ammonium sulfate (20.5% N); the phosphate fertilizer was superphosphate (12% P_2_O_5_); and the potash fertilizer was muriate potash (60% K_2_O).

### 4.2. Experimental Design and Rice Cultivation Management

The trial used a two-factor randomized block design. Three N fertilizer levels were applied: 210 kg ha^−1^ (conventional N, N3), 178.5 kg ha^−1^ (15% reduced N, N2), and 147 kg ha^−1^ (30% reduced N, N1). Two P levels were applied to each nitrogen level: 105 kg ha^−1^ (conventional P, P2) and 73.5 kg ha^−1^ (30% reduced P, P1), resulting in a total of six treatment groups (Table 3): N3P2 (conventional fertilization, CF), N3P1, N2P2, N2P1, N1P2, and N1P1. The N fertilizer for each treatment was applied three times: 36% as basal fertilizer, 24% as tiller fertilizer, and 40% as panicle fertilizer. P fertilizer was applied as basal fertilizer in a single application. Potassium fertilizer was applied at 105 kg ha^−1^ in two applications: 50% as basal fertilizer and 50% as panicle fertilizer.

Fertilizers were applied based on the leaf age. Basal fertilizer was applied one day before transplanting, tiller fertilizer at the 8.5-leaf stage, and panicle fertilizer at the 12-leaf stage. Each plot covered an area of 15 m² (3 m × 5 m) with three replicates. A 25 cm deep PVC partition was embedded in the ground between the plots to prevent water and fertilizer exchange. The seeding dates for the two years were 24 April and 28 April, whereas transplanting occurred on 25 May and 29 May, and harvesting occurred on 7 October and 11 October. Field management, including independent drainage and irrigation for each plot, along with weeding and pest control, adhered to high-yield and standard cultivation practices.

### 4.3. Sampling and Measurements

#### 4.3.1. Determination of Yield, Spikelets per Panicle, and Grain Weight

At rice maturity, three uniform growth points were selected in each plot. Effective panicles were assessed by examining 10 consecutive plant holes at each point to calculate the average number of effective panicles. Six holes were sampled based on the average number of effective panicles in each treatment group. The number of spikelets per panicle and 1000-grain weight were measured, and the number and weight of SGs and IGs were determined. SGs and IGs were classified as follows: each panicle was divided into upper, middle, and lower sections based on the number of primary pedicels. If the number of primary pedicels were divided into three, the sections were distributed equally. Otherwise, the distribution favored the lower section first, followed by the middle section. Grains directly attached to the primary branch in the upper section were considered SGs, whereas those connected to the secondary branch in the lower section were considered IGs. Four points of uniform growth were selected for each treatment, and two square meters of rice was harvested, threshed, dried, air-selected, and weighed. The actual yield was adjusted according to a standardized moisture content of 14.5% for *japonica* rice.

#### 4.3.2. Sampling and Determination of Grain Filling

At the onset of anthesis, 200 panicles of uniform size (5 cm from the leaf sheath) were selected from each plot. Fifteen panicles were randomly selected at 5-day intervals from anthesis to maturity. SGs and IGs were separated from each panicle, and unfertilized empty grains were discarded. The grains were then dried at 70 °C until a constant weight was achieved, after which they were hulled and weighed. The dynamics of grain weight gain were analyzed and modeled using the Richards equation, following the methodology outlined by Zhu et al. [64]:

$$W=\frac{A}{{\left(1+{Be}^{-kt}\right)}^{\frac{1}{N}}}$$

where W is the grain weight (mg), A is the ultimate grain weight (mg), t is the time after anthesis (d), and B, K, and N are the parameters of the equation.

Derivation of the Richards growth equation to obtain the grain-filling rate (G) curve:

$$G=\frac{{AKBe}^{-Kt}}{{N(1+{Be}^{-Kt})}^{\frac{\left(N+1\right)}{N}}}$$

Initial grain-filling potential (R_0_), maximum grain-filling rate (Gmax), time for maximum grain-filling rate (Tmax.G), grain weight with the maximum grain-filling rate (Wmax.G), mean grain-filling rate (Gmean), and active grain-filling period (D) were calculated from the parameters derived from the Richards equation:

$$R_{0}=\frac{K}{N}$$

$$Gmax=\frac{AK}{{\left(1+N\right)}^{\frac{\left(N+1\right)}{N}}}$$

$$Tmax.G=\frac{lnB-lnN}{K}$$

$$Wmax.G={A(N+1)}^{-\frac{1}{N}}$$

$$Gmean=\frac{AK}{2(N+2)}$$

$$D=\frac{2(N+2)}{K}$$

Based on the two inflection points of the grain-filling rate equation, G, the values of the two inflection points in the t-coordinate, denoted as t_1_ and t_2_, were obtained and combined with the actual final filling period t_3_ at 99% A to classify the early (<t_1_), middle (t_1_–t_2_), and late (t_2_–t_3_) stages of the filling process. The corresponding grain-filling durations and RGCs of the early, middle, and late stages were then calculated.

$$t_{1}=-ln(\frac{N^{2}+3N+N\sqrt{N^{2}+6N+5}}{2B})/K$$

$$t_{2}=-ln(\frac{N^{2}+3N-N\sqrt{N^{2}+6N+5}}{2B})/K$$

$$t_{3}=-ln(\frac{\left(\frac{100A}{99}\right)N-1}{B})/K$$

$${RGC}_{1}\left(earlystage\right)=\frac{W_{1}}{A}100\%$$

$${RGC}_{2}\left(middlestage\right)=\frac{W_{2}-W_{1}}{A}100\%$$

$${RGC}_{3}\left(latestage\right)=\frac{W_{3}-W_{2}}{A}100\%$$

where W_1_, W_2_, and W_3_ are grain weights at t_1_, t_2_, and t_3_, respectively.

#### 4.3.3. Measurement of Enzymatic Activities

Marked spikes of rice were collected from each plot every 5 d after anthesis. Initially, 10 spikes per collection were gathered until 15 d post-anthesis, which was reduced to five spikes per collection after 20 d. Each sample was wrapped in tin foil, flash-frozen in liquid nitrogen, and stored at −80 °C for later analysis. Before the enzyme activity measurements, the seeds were hulled. The activities of AGPP, GBSS, SSS, and SBE in SGs and IGs were tested using ELISA kits from Jiangsu Meimian Industrial Co., Ltd. (Jiangsu, China) [65,66]. However, the IG samples collected 5 d after anthesis were insufficient for starch synthase activity measurements. The amylase activity of SGs was tracked until 30 d post anthesis.

#### 4.3.4. Determination of Starch Content and Taste Value

The rice was ventilated and stored for three months. A 30.0 mg dry sample was added to 10.0 mL of distilled water and heated in a boiling water bath for 60 min. The extracts were centrifuged at 5000× *g* for 5 min, the supernatant was discarded, and the extraction was repeated three times. Subsequently, 2.0 mL of distilled water was added to the remaining precipitate, which was gelatinized in a boiling water bath for 15 min. After cooling, the sample was mixed with 2.0 mL of 9.2 mol L^−1^ HClO_4_ and shaken for 15 min, followed by adding 2.0 mL of 4.6 mol L^−1^ HClO_4_, and the mixture was vibrated for another 15 min. Post-extraction, 2.0 mL of distilled water was added, and the samples were centrifuged at 5000× *g* for 5 min. From the resulting solution, 4.0 mL of the supernatant was collected, and the volume was adjusted to 100 mL. Next, 1.0 mL of the extract was combined with 1.0 mL of distilled water and 5.0 mL of anthrone reagent (prepared by dissolving 2.0 g of anthrone in 1.0 L of concentrated sulfuric acid) for 2 min, and the absorbance at 620 nm was measured and applied to the standard curve to calculate the starch content. The amylose content was measured using the standard iodine colorimetry method in accordance with NY/T 2639-2014. The amylopectin content was calculated as the total starch content minus the amylose content. The taste value of the rice was evaluated using an STA1A rice taste meter (SATAKE, Saitama, Japan).

### 4.4. Statistical Analysis

Data were organized using Microsoft Excel 2019 and analyzed with IBM SPSS Statistics software version 22.0. Means were tested using Duncan’s test at a 0.05 probability level within the same year. Graphical presentations were created using Origin 2021 software. A structural equation model (SEM) was used to assess the impact of N and P levels on the grain weight and taste value of *japonica* rice utilizing Smart PLS (4.1.0.3) software.

## 5. Conclusions

Compared to conventional fertilization, the improved taste value of N2P1 was primarily attributed to an increased amylopectin/amylose ratio. Stable yields can be obtained by significantly increasing the spikelets per panicle and prolonging the grain-filling duration in IGs and in the middle and late stages of SGs, thereby maintaining grain weight. Increased nitrogen application improved the SSS and SBE activities in grains. In contrast, increased phosphorus fertilization enhanced the SSS and SBE activities in IGs but inhibited their activities in SGs. Appropriate reductions in nitrogen and phosphorus fertilizers prolonged the active grain-filling period but reduced the grain-filling rate and inhibited the activity of AGPP and GBSS in both SGs and IGs. Consequently, the amylose content was reduced, resulting in an improved taste. Low AGPP, SSS, and GBSS activities in SGs were the primary reasons for the low grain-filling rate and light grain weight, whereas the SSS, GBSS, and SBE activities regulated the grain-filling of IGs. High GBSS and SBE activities shortened the active grain-filling period. In conclusion, applying 178.5 kg N ha^−1^ of nitrogen in combination with 73.5 kg P ha^−1^ of phosphorus can achieve stable rice yields and enhance taste quality.

## Figures and Tables

**Table 1:** Effects of nitrogen–phosphorus interaction on grain-filling parameters of SGs and IGs.

Year	Treatment (N and P)		R_0_		Gmax (mg grain^−1^ d^−1^)		Wmax (mg grain^−1^)		Tmax.G (d)		Gmean (mg grain^−1^ d^−1^)		D (d)		R^2^	
			SG	IG	SG	IG	SG	IG	SG	IG	SG	IG	SG	IG	SG	IG
2021	N3	P2	0.816	0.143	1.316	0.959	10.38	10.93	9.66	24.52	0.893	0.630	29.11	31.66	0.994	0.991
		P1	0.400	0.140	1.145	0.899	10.09	10.56	11.49	25.27	0.775	0.591	30.24	32.68	0.997	0.982
	N2	P2	0.609	0.136	1.228	0.763	9.96	9.75	10.36	26.77	0.833	0.501	29.15	35.62	0.996	0.987
		P1	0.815	0.139	1.183	0.834	9.94	10.23	9.85	26.31	0.803	0.548	31.16	34.03	0.997	0.988
	N1	P2	0.579	0.129	1.075	0.635	9.31	8.17	11.90	28.76	0.729	0.420	31.18	36.79	0.955	0.991
		P1	0.653	0.133	1.083	0.665	9.40	8.35	11.33	27.06	0.735	0.442	31.64	36.75	0.993	0.993
2022	N3	P2	0.723	0.158	1.353	0.863	10.39	10.36	9.35	23.88	0.918	0.573	27.97	35.31	0.996	0.985
		P1	0.492	0.150	1.187	0.797	10.35	10.08	10.82	24.97	0.804	0.530	30.80	37.29	0.996	0.976
	N2	P2	0.512	0.145	1.336	0.661	10.35	8.93	10.41	25.48	0.904	0.441	27.09	40.43	0.999	0.992
		P1	0.703	0.147	1.338	0.714	10.24	9.45	9.63	25.50	0.908	0.475	27.76	39.15	0.999	0.989
	N1	P2	0.434	0.133	1.148	0.608	9.82	8.08	11.72	26.58	0.777	0.402	29.61	38.09	0.998	0.988
		P1	0.622	0.142	1.186	0.672	9.69	8.48	10.92	25.81	0.804	0.445	29.46	36.32	0.999	0.984

SG and IG represent superior and inferior grains, respectively; N3, N2, and N1 represent N application of 210, 178.5, and 147 kg ha^−1^, respectively; P2 and P1 represent P application of 105 and 73.5 kg ha^−1^, respectively. R_0_, initial grain-filling potential; Gmax, maximum grain-filling rate; Wmax.G, grain weight with the maximum grain-filling rate; Tmax.G, time for maximum grain-filling rate; Gmean, mean grain-filling rate; D, active grain-filling period.

**Table 2:** Effects of nitrogen–phosphorus interaction on the amylose and amylopectin content and taste value of rice.

Year	Treatment (N and P)		Amylose Content (%)	Amylopectin Content (%)	Starch Content (%)	Taste Value	Amylopectin/Amylose
2021	N3	P2	20.38 a	54.69 a	75.06 a	60.50 c	2.69 ab
		P1	19.80 ab	51.26 bc	71.06 c	58.00 d	2.59 bc
	N2	P2	20.13 a	52.20 b	72.32b c	62.50 b	2.60 bc
		P1	19.43 b	53.84 a	73.26 b	65.40 a	2.77 a
	N1	P2	19.33 b	48.97 c	68.30 d	56.88 d	2.54 c
		P1	19.15 b	50.62 c	69.77 cd	58.50 d	2.64 bc
2022	N3	P2	19.85 a	56.47 a	76.32 a	58.63 c	2.85 a
		P1	19.48 ab	51.10 b	70.58 bc	57.25 cd	2.62 b
	N2	P2	19.60 ab	52.03 b	71.63 bc	60.25 b	2.66 b
		P1	19.09 b	54.30 ab	73.39 b	62.13 a	2.85 a
	N1	P2	18.75 b	46.85 c	65.60 d	56.50 d	2.50 c
		P1	18.59 b	49.07 b	67.66 c	58.13 c	2.64 b
Variance analysis							
Nitrogen (N)			30.07 **	52.23 **	75.00 **	67.35 **	9.70 **
Phosphorus (P)			17.79 **	0.18 ^ns^	2.19 ^ns^	3.40 ^ns^	3.30 ^ns^
N × P			1.73 ^ns^	27.49 **	29.28 **	12.61 **	15.37 **

Different letters indicate statistical significance at the *p* < 0.05 level between different treatments in the same year. ** indicates that the effects of variables are significant at 0.01 levels; and ns indicates no significant effect. N3, N2, and N1 represent N applications of 210, 178.5, and 147 kg ha^−1^, respectively; P2 and P1 represent P applications of 105 and 73.5 kg ha^−1^, respectively.

**Table 3:** Amount and period of fertilization for each treatment (kg ha^−1^).

Treatment		Total Amount of Fertilizer		Base Fertilizer		Tillering Fertilizer	Panicle Fertilizer
		N	P	N	P	N	N
N3	P2	210.0	105.0	75.60	105.0	50.40	84.0
	P1	210.0	73.5	75.60	73.5	50.40	84.0
N2	P2	178.5	105	64.26	105.0	42.84	71.4
	P1	178.5	73.5	64.26	73.5	42.84	71.4
N1	P2	147.0	105.0	52.92	105.0	35.28	58.8
	P1	147.0	73.5	52.92	73.5	35.28	58.8

## Data Availability

The data used to support the findings of this study can be made available by the corresponding author upon request. The data are not publicly available due to the fund information and other papers not having been published; these data will not be uploaded.
